# Beliefs About Parent Participation in School Activities in Rural and Urban Areas: Validation of a Scale in Mexico

**DOI:** 10.3389/fpsyg.2020.00639

**Published:** 2020-04-24

**Authors:** Sonia Beatriz Echeverría-Castro, Ricardo Sandoval-Domínguez, Mirsha Alicia Sotelo-Castillo, Laura Fernanda Barrera-Hernández, Dora Yolanda Ramos-Estrada

**Affiliations:** Department of Psychology, Instituto Tecnológico de Sonora, Ciudad Obregón, Mexico

**Keywords:** parental involvement, beliefs, parents, confirmatory factor analysis, invariance

## Abstract

The objective of this study was to test a measurement and invariance model for a scale of beliefs about parent participation in school education for children residing in both rural and urban areas. The questionnaire was answered by 2,576 parents, 52% from urban areas and 48% from rural; also an exploratory confirmatory multigroup analysis was performed to identify invariance. The final version of the instrument consisted of two factors with three items each, showing a goodness of fit, in addition to adequate indices. The invariance analyses indicated that both samples were equivalent in structure and factorial weight. The comparative fit index was greater than 0.95 for each model, and when compared with the restrictive model, the differences were less than 0.01; therefore, the instrument is considered applicable.

## Introduction

Parents play important roles in their children’s learning process and in the regulation of their behavior at school; likewise, parents can also be support agents for the school and for educational outcomes (Valdés and Urías, 2010). Despite this, studies report a low involvement of parents in all school settings, from supporting children doing their homework to the school-parent relationship, including communication with teachers or principals (Valdés et al., 2009).

One of the main personal psychological motivators for parents to become involved and participate in the academic activities of their children consists of the beliefs they have about their roles as parents, getting involved in activities they consider as their responsibility and leaving the rest to the teachers or the school (Walker et al., 2011). Hoover-Dempsey and Sandler (2012) call this variable the construction of the parental role; these authors also consider it a basis for their model of the parental involvement process.

Hoover-Dempsey et al. (2005) propose a model by levels of influence. In the first level, parents are involved through: (a) beliefs about the parental role regarding what they consider their responsibility doing at their children’s school; (b) self-efficacy to support their children doing their homework and respond to the invitations of their children and the teachers. In level two, contextual variables such as time and energy, knowledge and skills for involvement are added. The third level refers to the mechanisms of participation:behavior modeling, motivation, instruction, and reinforcement. The fourth level includes student perceptions of their parents’ actions apropos of the previous level, their self-efficacy to learn and their relationship with teachers. Finally, in the fifth level, the student academic performance is explained.

The goal of this study is to test an instrument for measuring the beliefs of parents concerning what they should do to support their children at school environments as part of their parental role; such a tool is relevant because beliefs are the main motivators in the decision of parents to become involved in their children’s school (Hoover-Dempsey et al., 2005). In general, parents who believe that they should have an active role in the education of their children are more likely to participate than are those who think that parent involvement is not necessary or that it is better not to intervene (Green et al., 2007). Deslandes and Bertrand (2005) explain that it is more frequent for parents to become involved in school activities at home when they believe it is their responsibility to do so, and especially when such belief is accompanied by invitations from their children to participate.

The beliefs parents have in relation to the roles they should assume in their children’s education are developed through their own experience within the groups in which they have become socialized, such as family, school and community; this helps to establish their role beliefs, which affect their involvement behaviors in their children’s school life and in their interactions at home as to school-related tasks, projects and other activities for which students ask for their parents participation. In general, these personal beliefs parents have about the goals of school education and their own role in it influence their involvement (Hoover-Dempsey et al., 2004).

There are diverse parents mindsets with respect to the objectives of education, their own involvement in educational centers, and their role in this process. Some parents believe that the school is responsible for ensuring the success of their children and that parent participation is not necessary, while others perceive a shared responsibility between the school and the family; the latter are the most willing to participate by supporting their children at home and at school (Reed et al., 2000).

Walker et al. (2005) present three types of role construction centered on parents: parent-focused, which reflects the beliefs and behaviors of parents, where the parent is ultimately responsible for the education of the child; school-centered roles, which reflects the beliefs and behaviors of parents where the school is ultimately responsible for the child education; and, finally, the partnership-focused role, which reflects beliefs and behaviors where parents and schools together are responsible for the education of the child.

Two qualitatively different aspects can be identified on the topic of parents participation in the education of their children: the first is related to parental engagement with the school, for example, communication with teachers and the principal, participation in events and activities organized by the school, and involvement in parents associations; the second refers to the learning support parents provide at home, for example, assist in doing homework; in doing so, they help their children to develop favorable attitudes toward school and they also create conditions for students to learn (Sánchez and Valdés, 2014).

Walker et al. (2005) developed an instrument for parental role beliefs, a nine-item scale with six response options that aims to measure two types of assistance: supporting the child and supporting the school. These are some examples of the items: “It is my responsibility to talk with my child about his or her school day,” “I believe that every parent is responsible for what is happening in school.”

Urban and rural environments create different school contexts, therefore, parents have different experiences and perceptions about schools and the educational objectives aimed. Rural schools are often associated with indigenous and/or farming populations. Villarroel and Sánchez (2002), when comparing rural and urban areas, find that parent participation is similar, but there is a significant predominance of participating mothers in rural areas.

Azaola (2010) notes that in rural areas, mothers care for their children with regard to school affairs, and fathers do not consider it to be their job. Additionally, in rural environments, children get little support for solving complicated tasks or studying for exams, as parents do not feel capable of providing such help because of their own lack of education; besides, communication regarding what happens at school is scarce, since children talk little with parents about what happens at school and do not tell them when they have tests. In her study on rural communities of a central region in Mexico, Azaola (2010) notes that parents compensate the lack of intellectual and economic support for their children academic development by providing discipline and emotional support. In urban areas, this is a little different: there is a higher level of schooling among the population, and fathers do consider that academically supporting their children is their job, because more women financially contribute to the household, particularly in the early years of life and when boys and girls are at school age (Sandoval et al., 2017).

Durston (1995) highlights that cultural differences of rural areas with respect to language, thinking styles and expressions complicate learning, especially when teachers do not use what is already familiar to students; as the author states, a new school culture of communication and relationships should emerge from the interactions between the actors involved, mainly teachers and parents. Many teachers of rural schools are culturally deprived, and this creates a distance with parents instead of an assimilation process; in urban schools, at least in those attended by boys and girls with better living conditions and parents with higher education levels, teachers feel more comfortable and support parental beliefs about participating in the education of their children. This is in particular evident within areas where there are more conflicts at school environments. Of note are the fewer opportunities that children in rural areas have accessing goods, from the most basic needs to up-to-date technology, including computer equipment and internet; situations often resulting from the parents low education levels (Paes de Barro et al., 2009). Roemer and Ünveren (2017) remark that parental education levels, especially those of mothers, and occupations are indicators of inequity in the access to opportunities.

Given that beliefs are important for parental involvement in school activities, it is important to understand the existing differences and which ones of these can impact the measurement model posed in this document; as well as those differences affecting the results and interpretation. Because of the importance of beliefs in parental involvement, an instrument that allows an understanding of those beliefs, applicable to different schooling levels and parents cultural capital, suitable for urban and rural areas, is necessary for decision-making that favors the family-school relationship in different contexts. It is also important to review the relevance of instruments and their psychometric properties in different contexts. Therefore, the objective of this study was to test a measurement and invariance model of a belief scale for parent participation in their children’s school education, within both rural and urban areas.

## Materials and Methods

This is an instrumental study (Carretero-Dios and Pérez, 2005; Ato et al., 2013) in which the psychometric properties of a scale of beliefs about parent participation in their children’s school education were obtained, as was the validity of the measurement model for parents residing in urban and rural areas.

### Participants

The instrument was completed by 2,576 parents with children in basic education schools within either urban or rural areas. The sample was selected by convenience, taking into account the total population of elementary students in Sonora, a Mexican northwestern state that borders the United States of America. Rural populations are mainly located in the south of the state; the rural sample was taken from four towns located on this area. On the other hand, urban sample was taken from the four largest cities of the state.

For the analysis, random smaller samples (20%) were obtained from the general sample, in order to ensure suitable samples in line with both the model and the methods employed (Jackson, 2003). The subsample used to run the model consisted of 52% of parents residing in urban areas [95% CI 48–56 bootstrap%] and 48% of parents residing in rural areas [95% CI 46–49 bootstrap%]. For the urban areas, 40% were fathers and 60% were mothers; for the rural areas, 58% were mothers, and 42% were fathers. The four rural towns considered in this study fit in the rural population classification made by the National Institute of Statistics and Geography (INEGI, 2010): less than 2,500 inhabitants, limited urban services, no paved street (or just the main street), agriculture is the leading economic activity.

Parents’ age information was removed because of a high percentage of missing values (up to 12%). No significant differences or correlations were observed between the presence of other children in elementary school or other educational levels and the participation of parents (supporting child and school) in urban and rural areas (see Annex 1). Mothers in rural areas had lower levels of education. In rural areas, a higher percentage of mothers reached only primary school education, and a lower percentage got university education; for fathers, the results were similar, but the differences were slightly greater between those who resided in cities (urban) and those who resided in towns (rural) (see Table 1).

**TABLE 1:** Education level of the parents in urban and rural areas (percentages).

	**Mothers**		**Fathers**	
		
	**Urban (%)**	**Rural (%)**	**Urban (%)**	**Rural (%)**
Elementary school	3	11	3	16
Middle school	30	40	28	38
High school	40	30	35	28
University	27	19	34	18

The referred existing learning opportunities differences between urban and rural families are: access to books, access to internet. A comparison of these scenarios within the urban and rural contexts of the region of interest is presented below (see Table 2). Access to a greater number of educational institutions near of their homes is most likely an important factor for students’ educational progression; it is worth noticing that all the rural areas considered in the sample of this study were located at almost an hour from educational institutions, and had poor public transportation services.

**TABLE 2:** Available sources of learning support for students in urban and rural areas (percentages).

	Urban (%)	Rural (%)
Computer access at home	65	30
Internet access at home	57	21
Books available at home (approximately 10 books)	20	13

On average, urban families had four members and rural families had five. The daily income per urban family was 20 United States, 5 dollars per member; in the rural family, it was 10 United States, 2 dollars per member. The most frequent occupations were similar for urban and rural fathers with unskilled jobs, especially in the rural area. In the case of mothers, predominated the unpaid work of housekeeping; a distinguishing fact is that women in the rural area had the highest percentage of skilled work, beyond that of fathers in rural or urban, and mothers in urban areas (see Table 3). This information reveals the two different realities experienced by the families that live in such areas.

**TABLE 3:** Type of occupation parents in urban and rural areas have (percentages).

	Mothers		Fathers	
		
	Urban (%)	Rural (%)	Urban (%)	Rural (%)
Housewife (unpaid)	48	63	0	0
Not qualified job	3	7	59	73
Qualified job	38	18	17	10
Self-employed professional	9	9	12	8
Retired	0	0	1	1
Executive	0	0	2	0
Not present	2	3	9	8
Total	100	100	100	100

### Instrument

The instrument consisted of two parts, the first one with items aimed to obtain information of sex, age, number of child attending school, family’s learning context aspects, and economic situation. Also, two general questions were added to ask: if parents believed that their participation influenced their children’s school success; and about the time they spent supporting their children in educational matters.

The requested information on the learning contexts was on these subjects: (a) parents’ educational levels, (b) computer at home, (c) internet at home, and (d) books at home. The economic situation indicators were: (a) average family income per day, (b) number of people who support themselves with said income, and (c) kind of occupation. The kind of occupation referred to labor activities for which an income was received; although it does not imply a payment, domestic labor (taking care of children) was also included. Unskilled jobs, meanwhile, were those occupations that only require brief training; the qualified were those requiring a degree of specialization and received higher remuneration than the non-qualified. Another category of professionals was included for self-employees, retirees and parents in executive jobs (these are responsible for decision-making and generally have higher salaries than people in the other categories). These job categories are simplifications of those included in the National Labor Classification System [Sistema Nacional de Clasificación del Trabajo] (INEGI, 2018).

The one-dimensional scale of beliefs about parent participation in school activities of their children developed by Walker et al. (2005) was applied in its original version with 10 items, in a continuum: from active (partnership oriented and with high score), to passive (with lower score). Two factors of the scale were tested in this study: (a) supporting children in their school activities, and (b) supporting the school. The instrument was answered using a scale with five values ranging from strongly disagree (1) to strongly agree (5). In its original version, the response scale had six response options, however, the options were reduced to five in order to facilitate understandability (a pilot test indicated confusion with the six-option version). For the original version, Cronbach’s alpha was 0.816, but validation of the measurement model was not reported. For this instrument, Lavenda (2011) reported a measurement model with an adequate fit, reporting normed fit index (NFI) and comparative fit index (CFI) values higher than 0.90, a root mean square error of approximation (RMSEA) of 0.06 and invariance for samples of Jewish and Arab parents. As part of one study with Mexican population, an adequate model fit with a two-dimensional structure was reported (Sandoval et al., 2017).

### Procedure

The instrument, originally written in English by Walker et al. (2005), was adapted to Spanish using a cross-translation. Then, was revised a version of the same scale in Spanish, presented by Hoover-Dempsey et al. (2005), which coincided with the Spanish version from the aforementioned instrument obtained by cross-translation. A panel of experts consisting of three specialists determined the content validity; two items were reworded due to redundancy. There was 100% agreement among the reviewers regarding editorial adjustments and the elimination of one item; but the full original scale was applied, that item was removed later during the different analyzes. The instrument was further adapted by adding two items to include aspects of school coexistence, which is a topic related to respect among classmates and required to be addressed by teachers and parents in Mexican schools (“Teaching my child to get along with children” and “Teaching my child how to coexist peacefully”).

Once the instrument was finalized, it was applied. Before applying the questionnaire, authorizations from the different elementary education institutions principals were requested; from the beginning, it was made clear that parents had the option to reject participating in the study. Next, after explaining the objectives of the project to the teachers, their signatures indicating consensus support were requested. After gaining teacher support, each child was asked to deliver his or her parents an invitation to respond the instrument; prior informed consent from the parents was obligatory in order to complete the questionnaire. The instrument was sent to the parents through their children in an envelope; teachers supported this study by delivering the envelopes to the students. Finally, the parents answered the questionnaires at home and returned the sealed envelopes to the teachers.

### Data Analysis

The SPSS statistical package was used to perform exploratory factor analysis (EFA) with maximum likelihood extraction (to reduce the effect of the normality requirement), and varimax rotation; AMOS was used to perform confirmatory factor analysis (CFA), and determine multigroup invariance. For the EFA, the general sample was used; followed by parents from urban areas and then by parents from rural areas. The distribution of the data for each of the variables had a guaranteed asymmetry between +1, −1, and a kurtosis of 0.6 or less (Lloret-Segura et al., 2014).

The same orthogonal rotation criteria were considered using varimax in agreement with those used for the original instrument (Hoover-Dempsey et al., 2005; Walker et al., 2005), noting that rotation was also performed obliquely using oblimin, and that the results were very similar, without differences that would reveal the necessity of a modification. In the case of extraction, maximum likelihood was used to decrease the parametric requirement, in addition to the use of a bootstrap technique to compensate for this requirement of normality. To verify the suitability of the sample for EFA, the Kaiser-Mayer-Olkin coefficient (KMO) and the Bartlett test of sphericity were used.

Returning to these considerations, once one of the items got eliminated because of tis low factorial weight, CFA was performed using the variables that had previously met the requirements for this analysis from the Pearson correlation matrix with regressions to determine which items (variables) should be incorporated into the CFA. The use of structural and bootstrap equations in AMOS allowed the validation of model fit in other subsamples.

Reliability was determined using Cronbach’s alpha and, although it was possible requirements of the first could not be met, coefficient omega was used to corroborate reliability (Dunn et al., 2014); this corroboration involved following the formula and procedure, using Excel and the data obtained from the EFA (Ventura–León and Caycho-Rodríguez, 2017). The composite reliability and average variance extracted (AVE) and the square root of the AVE showed that the correlation with other constructs were used to obtain evidences of convergent and discriminant validity (Fornell and Larcker, 1981).

### Ethics Statement

Institutionally, an ethics committee approved the study protocol before research commenced. At all times, the participants were entitled to refuse to participate, including the principals, teachers and parents invited to complete the instrument. Feedback was provided through educational material designed for families, that is, a brochure with a magnet to place on the refrigerator (following the customs in this region of using the refrigerator to display children’s documents); it was sent to the families through the principals of the participating schools.

## Results

It was observed that a similar percentage of parents responded that their participation in children’s school affairs influences their learning; 51% indicated that it has no effect and 49% considered that it does contribute to the good academic performance of their children. When contrasting by area and gender of the parent, no significant differences were found.

Regarding time available to attend to their children’s school affairs, as included as a variable in Hoover-Dempsey model, the parents in both regions dedicated similar amounts of time, and most believed that parent participation is appropriate (78% of parents residing in rural areas and 70% of parents residing in urban areas), with a significant difference between them (*X^2^* = 14.93, *gl* = 2574, *p* = 0.01); that is, a higher percentage of parents in rural areas feel comfortable with the time they invest on caring for their children apropos of education. The differences in access to opportunities lead to, in addition to CFA, the inclusion of a multigroup for the review of rural-urban invariance.

### Convergent and Discriminant Validity

The composite reliability ranges were 0.83 (support child) and 0.78 (support school). The factor loads was up the 0.68–0.85 and AVE of the model was 0.63 and 0.54 (support child and school, respectively), which indicates the presence of convergent validity. The square root of the AVE of the construct was higher (up to 0.7) than the correlation with other constructs (lower than 0.60), thus verifying the discriminant validity (Fornell and Larcker, 1981).

### Exploratory Factor Analysis

First, EFA was performed for the original scale with 10 items plus the two items added during the adaptation. The KMO value was 0.866, and the Bartlett test of sphericity result was 0.00 with a chi-square value of 7677. The factorial structure of the two factors was the same as the original. The first refers to the activities that parents believe they should support, and the second refers to the support they believe they should provide to the school. Two items were eliminated because they had factorial weights lower than 0.30 and were in two factors. The support at home factor had an internal consistency of 0.85, and the support at school factor had an internal consistency of 0.77; the alpha for the total scale was 0.83, and the model explained 53% of the variance. Subsequently, EFA was performed for each sample (rural and urban), and the structure of the two factors was verified.

### EFA on Urban Area

A KMO value of 0.801 was obtained, the instrument had a total explained variance of 72.57%, and the items were grouped into two factors with four items each: (a) support for children at home, showing reactive factorial weights between 0.850 and 0.940, with a variance of 37.69%; and (b) support to school, with factor weights oscillating between 0.832 and 0.783, with a variance of 34.88%.

### EFA on Rural Area

The KMO value of 0.789 was acceptable, with a total explained variance of 68.31%, and a two-factor structure with four items each. For the first factor, support for the child with his or her school-related tasks for home, the factorial weights were between 0.858 and 0.777, with an explained variance of 35%. For the school support factor, the factor weights ranged between 0.799 and 0.781, with an explained variance of 33.23%.

### Confirmatory Factor Analysis -Multigroup- Invariance

In the CFA, six items were grouped into two factors, maintaining the two-factor structure and eliminating two items since they were substantially reducing the goodness of fit of the original model. The CFA of each sample indicated that the model measuring two factors with three items each was acceptable for both cases (parents in urban and rural areas). The comparative goodness of fit index (CFI), considered one of the main indices for these cases, had values above 0.90 (Cheung and Rensvold, 2002; Elosua, 2005), and RMSEA values were less than 0.05. According to the measures of incremental adjustment and parsimony, these values were significantly higher than those for the independent model and very similar to those for the saturated model (Ruiz et al., 2010; Table 4).

**TABLE 4:** Absolute, incremental and parsimony indices for the generated models, confirmatory factor analysis for urban and rural areas (*p < 0.05).

Model	Absolute indices			Incremental indices			Parsimony indices	
			
	*X*^2^	GFI	RMSEA	AGFI	TLI	CFI	CMIN/DF	AIC
**Factor solution for the urban area**								

Independent	3021.2	0.997	0.389	0.296	0.000	0.000	201.416	3033.24
Saturated	0.000	1.00						42.000
2 factors	10.406	0.497	0.015	0.993	0.999	0.999	1.300	36.401

**Factor solution for the rural area**								

Independent	2268.0	0.545	0.347	0.364	0.000	0.000	151.201	2280.01
Saturated	0.000	1.00						42.000
2 factors	19.016	0.995	0.033	0.987	0.991	0.995	2.377	45.016

*GFI, (Adjusted) Goodness of Fit; RMSEA, Root Mean Square Error of Approximation; AGFI, Adjusted Goodness of Fit Index; TLI, Tucker Lewis index; CFI, Comparative Goodness of Fit Index; CMIN/D, Chi-Squared Ratio ff Degrees Of Freedom; AIC, Akaike Information Criterion.*

In the CFA, in both samples, the items saturated adequately, revealing moderate correlations between the factors (see Table 5), and an acceptable coefficient omega for each factor (Ventura–León and Caycho-Rodríguez, 2017; Table 6).

**TABLE 5:** Confirmatory factor analysis results for both samples.

	Support the child		Support the school	
		
Items	Urban	Rural	Urban	Rural
Support my child in understanding his/her homework.	0.850	0.839		
Help my child with his/her homework.	0.776	0.690		
Teach my child to live peacefully.	0.765	0.717		
Making the school better.			0.806	0.754
Speak with other parents about the school.			0.704	0.704
Ensure that the school has what it needs.			0.699	0.647

Correlations between factors				

Support the child	–	–		
Support the school	0.55	0.56	–	–

**TABLE 6:** Goodness of fit indices for each of the models tested for factorial invariance (*p < 0.05).

Model	Fit indices						
	
	*X*^2^	g	CMIN/DF	NFI	CFI	RMSEA	AIC
Model without restrictions	29.420	16	1.839	0.994	0.997	0.018	105.420
Metric invariance	43.239	20	2.162	0.992	0.996	0.021	111.611
Strong factorial invariance	80.397	29	2.772	0.985	0.990	0.026	130.397

*CMIN/D, Chi-Squared Ratio of Degrees of Freedom; NFI, Non-Normed-Fit Index; CFI, Comparative Goodness of Fit Index; RMSEA, Root Mean Square Error of Approximation; AIC, Akaike information criterion.*

The indices obtained (Table 6) showed the equivalence of the basic measurement models between rural and urban areas; that is, there is invariance of the factorial structure between the two. Although the chi-squared value is high, the rest of the indices point to the similarity of both models, enough to accept the hypothesis of invariance (NFI greater than 0.9; CFI greater than 0.95; RMSEA lower than 0.05) (Elosua, 2005).

In the case of metric invariance by placing restrictions on the factorial loads on the base model, the general fit index (GFI) and the RMSEA indicated equivalence. Additionally, the CFI and Akaike results did not show relevant differences, although the values slightly increased with respect to the restricted model (see Table 6).

When assessing the strong factorial invariance (intercept) through the independent model and the model with nested metrics, the CFI of the models, other than the non-restrictive model, were less than 0.01; the difference in the CFI for metric invariance was 0.001, and that for strong factorial invariance was 0.007 (Cheung and Rensvold, 2002). The results of these estimations allow establishing that the two belief models for the parents who reside in rural and urban areas are equivalent with respect to the factor coefficients, as well as the strong or intercept coefficients (see Table 7).

**TABLE 7:** Coefficient omega for the factors obtained.

Factor	Urban	Rural
	
	ω	ω
Support the child	0.939	0.886
Support the school	0.898	0.846
Total	0.915	0.874

According to the results obtained for each sample, the instrument had an acceptable coefficient of omega reliability in general, as well as for each one of the factors (see Table 7). The values of reliability (alpha) were also above 0.80; the omega coefficient verified the reliability (Revelle and Zinbarg, 2009; Peters, 2014). The scale in Spanish version is presented in Annex 2, with evidences of reliability, convergent and discriminant information.

## Discussion

The differences found when comparing rural to urban contexts, on the subject of opportunities to access information sources, revealed the relevance of identifying invariances in the instrument for measuring beliefs about the participation of parents in children’s school education. In the multigroup CFA, the model fit adequately with two factors containing three items each, which is consistent with the bifactorial structure proposed by Hoover-Dempsey et al. (2005). The initial proposal consisted of 10 items, but this number was reduced to three per factor. This number of items is the minimum acceptable number; in the future, in other studies, items for these indicators should be written and tested to improve the instrument. Although the number of items is small, when this kind of brief instruments is answered, the acceptance rates are higher and are answered with greater caution, since the probability of fatigue is reduced, leaving fewer unanswered questions than when questionnaires are extensive.

The configurational invariance results indicate that the instrument is applicable for rural and urban samples, with consistency between the structure and factorial weights, with an instrument with two factors suitable for parents in both rural and urban areas. This instrument can be applied in schools in both areas; it is very short, making it easy for parents to respond, in contrast to low recovery rates characteristic of instruments that are extensive. The context of opportunities, while different in each area, maintains the variables for measuring invariance of the measurement model, with adequate fit with a two-factor structure for parents residing in both areas.

For reliability, the coefficient omega results were similar to those reported by Walker et al. (2005), who found an internal consistency of 0.81 (with Cronbach’s alpha) for questionnaire that measures beliefs regarding parental roles in children’s school activities.

The scale for beliefs regarding parent participation at school is appropriate for implementation in the northwestern area of Mexico, in addition to being brief and easily understood by parents in rural areas with an education level lower than those of parents in urban areas. Consistency with the model proposed by Hoover-Dempsey et al. (2005) was maintained. This brief scale provides the option of applying a short instrument for use in the Mexican context, characterized by a diversity and breadth of schools in rural and urban areas.

It is necessary to mention several limitations of this study: first, since the participants lived in the northwestern region of the country, it is not possible to conclude that a representative sample of the Mexican population was used; furthermore, the present research did not consider the measure of other variables to test criterion validity. Despite these limitations, this questionnaire identifies role’s beliefs about parental involvement in their children’s school activities, which, in brief, is rewarding as it allows parents with different educational levels and reading habits to respond easily. The duration of about 5 min to answer makes parents more willing to respond. It makes it possible to teachers and principals to know the parents’ beliefs about their responsibility regarding the children’s academic succeed. Teachers can evaluate and discuss with parents the value of their involvement in both their child’s and school’s support. The matter is truly important because the data reveals that the actual behaviors of participation rates are reduced, especially in those related to the school attention around the country (Valdés and Urías, 2011).

## Data Availability Statement

The datasets generated for this study are available on request to the corresponding author.

## Ethics Statement

The studies involving human participants were reviewed and approved by Ethics committee of Instituto Tecnológico de Sonora. The patients or participants provided their written informed consent to participate in this study.

## Author Contributions

SE-C designed the project. RS-D and DR-E performed the data collection, SE-C and MS-C analyzed the data and supervised the findings of this study. SE-C and LB-H wrote the manuscript.

## Conflict of Interest

The authors declare that the research was conducted in the absence of any commercial or financial relationships that could be construed as a potential conflict of interest.

## Annex

**Annex 1:** Number of children in family.

Have children in primary or other educational levels	Type of area	
	
	Urban%	Rural%
Other child in kindergarten	18	38
Other child in middle school	40	23
Only one child in elementary school	2	0
Have two children in elementary school	26	23
Have three children in elementary school	3	5
Have four or more children in elementary school	1	1
Total	100	100
		

**Annex 2:** Parental Role Construction for Involvement in the Child’s Education Scale - Spanish version.

Factor	Items	λ	CR*	AVE
Apoyo al niño/Support for the	Apoyar a mi hijo a que entienda sus tareas.	0.85	0.84	0.631
child	Ayudar a mi hijo con la tarea.	0.77		
	Enseñar a mi hijo a convivir pacíficamente.	0.76		

Apoyo a la escuela/	Hacer que la escuela mejore.	0.80	0.77	0.54
Support for the school	Hablar con otros padres de familia de la escuela.	0.70		
	Asegurarme de que la escuela tenga lo que necesita.	0.69		

*CR^∗^, Composite reliability; AVE, Average Variance Extracted.*
